# Prevalence and factors associated with microvascular and macrovascular diabetes complications in adult Ugandans: A systematic review and meta-analysis

**DOI:** 10.1371/journal.pone.0312792

**Published:** 2026-06-08

**Authors:** Davis Kibirige, Ronald Olum, William Turyamureeba, Bethan Morgan, Andrew Peter Kyazze, Yakobo Nsubuga, Jerom Okot, William Lumu, Felix Bongomin

**Affiliations:** 1 Department of Medicine, Uganda Martyrs Hospital Lubaga, Kampala, Uganda; 2 Non-Communicable Diseases Program, Medical Research Council/Uganda Virus Research Institute and London School of Hygiene and Tropical Medicine Uganda Research Unit, Entebbe, Uganda; 3 School of Public Health, College of Health Sciences, Makerere University, Kampala, Uganda; 4 Manchester University NHS Foundation Trust, Manchester United Kingdom; 5 Department of Medicine, College of Health Sciences, Makerere University, Kampala, Uganda; 6 Faculty of Medicine, Gulu University, Gulu, Uganda; 7 Department of Medicine, Mengo Hospital, Kampala Uganda; 8 Department of Medical Microbiology & Immunology, Faculty of Medicine, Gulu University, Gulu, Uganda; 9 Department of Internal Medicine, Gulu Regional Referral Hospital, Gulu, Uganda; National Healthcare Group, SINGAPORE

## Abstract

**Introduction:**

The rising prevalence of diabetes in Uganda has led to a disproportionate increase in the burden of diabetes complications. We conducted a systematic review and meta-analysis to document the prevalence and factors associated with five chronic diabetes complications in adult Ugandans with diabetes.

**Methods:**

We searched Medline, EMBASE, CINAHL, Cochrane Library, and Africa Journal Online databases from 25^th^ May 2024–29^th^ May 2024. We included studies reporting information on the prevalence and factors associated with the five chronic diabetes complications. We conducted a random-effect meta-analysis to determine the pooled prevalence of each diabetes complication. Using narrative synthesis, we described the factors significantly associated with each diabetes complication.

**Results:**

A total of twenty studies reporting information on 11,400 participants were considered. The pooled mean (standard deviation) age of the participants was 54.8 (3.6) years.

For the microvascular diabetes complications, the pooled prevalence of diabetic neuropathy, retinopathy, and nephropathy was 56.8% (95% CI 44.9–68.7, I^2^ = 98.56%, p < 0.001), 19.5% (95% CI 3.9–35.2, I^2^ = 99.60%, p < 0.001), and 17.7% (95% CI 7.3–28.0, I^2^ = 99.36%, p < 0.001), respectively. For the macrovascular diabetes complications, the pooled prevalence of peripheral arterial disease and diabetic foot disease was 32.2% (95% CI 15.8–48.7, I^2^ = 97.67%, p < 0.001) and 5.5% (95% CI 1.7–9.2, I^2^ = 90.22%, p < 0.001), respectively.

Hypertension comorbidity, physical inactivity, family history of diabetes, body mass index ≤30 kg/m^2^, and pregnancy were associated with diabetic nephropathy in three studies. In two studies, a history of a foot ulcer and age > 60 years were associated with diabetic neuropathy, while female sex, hypertension comorbidity, and use of glibenclamide were associated with peripheral arterial disease.

**Conclusions:**

This study demonstrates a high prevalence of chronic diabetes complications in adult Ugandans with diabetes. Timely screening and optimal management of chronic diabetes complications should be encouraged in Uganda.

## Introduction

Globally, there is an increasing burden of diabetes. According to the most recent International Diabetes Federation (IDF) estimates, about 24 million (1 in 22) adult Africans aged between 20 and 79 years live with diabetes [1]. Additionally, among the IDF regions, Africa has the highest proportion of people with undiagnosed diabetes (54%) [1]. Because of the high rates of undiagnosed diabetes in Africa, coupled with low access to essential diabetes medicines and diagnostics and poorly structured healthcare systems, the prevalence of chronic diabetes-related complications in adult Africans with diabetes is considerably high [1–3].

Chronic hyperglycaemia is associated with several chronic microvascular and macrovascular complications, such as diabetic retinopathy, neuropathy, nephropathy, diabetic foot disease, peripheral arterial disease, and stroke [4]. These complications develop due to hyperglycaemia-induced increase in oxidative stress, formation of advanced glycation end-products, and pro-inflammatory state [4].

Uganda has documented a two-fold increase in the prevalence of diabetes and prediabetes over the last ten years [5,6]. Currently, the proportion of adult Ugandans who are unaware of their diabetes status is still relatively high [6]. Furthermore, access to affordable essential diabetes medicines and diagnostics remains challenging [7–9]. Due to the high rates of undiagnosed diabetes and poor access to essential diabetes medicines in Uganda, the prevalence of chronic diabetes complications in adult Ugandans with diabetes continues to increase steadily.

Several observational studies have been conducted in Uganda to document these chronic diabetes complications. The limitation of most of these studies is the significant heterogeneity in the reported prevalence due to the differences in the study diagnostic approaches, settings, and designs. It is important to understand the true prevalence of the different diabetes complications by pooling the results of locally performed studies. Identifying the factors associated with chronic diabetes complications would be important in guiding the implementation of precise and practical approaches to prevent and manage these chronic diabetes complications in Uganda.

To understand the true prevalence and factors associated with chronic diabetes complications in Uganda, we conducted a comprehensive systematic review and meta-analysis to document the pooled prevalence and factors associated with the five chronic diabetes complications in adult Ugandans with diabetes.

## Methods

This systematic review and meta-analysis was conducted according to the criteria outlined in the Preferred Reporting Items for Systematic Reviews and Meta-Analyses (PRISMA) statement [10] (**S1 Table**). The study protocol was registered in the PROSPERO International Prospective Register of Systematic Reviews (CRD42024541251).

### Search strategy

With the help of a librarian (BM), we searched PubMed, EMBASE, CINAHL, Cochrane Library, and Africa Journal Online databases for published studies from 1946 to 2^nd^ May 2024. The data search was conducted from 25^th^ May 2024–29^th^ May 2024.

The following search terms were used “Diabetes mellitus” OR ‘’diabetes mellitus type 2” OR “diabetes” OR “diabetes mellitus type 1” OR “diabetes mellitus type 2” OR “type 2 diabetes” OR “type 2 diabetes mellitus” OR “type 1 diabetes” OR “type 1 diabetes” OR “type 1 diabetes mellitus” OR “diabetic” OR “type 2 diabetic” OR “type 1 diabetic” AND “microvascular complication” OR “macrovascular complication” OR “microvascular diabetic complication” OR “macrovascular diabetic complication” OR “microvascular diabetes complication” OR “microvascular diabetes complication” OR “microvascular” OR “macrovascular” OR “chronic diabetes complication” OR “diabetic retinopathy” OR “retinopathy” OR “maculopathy” OR “diabetic nephropathy” OR “nephropathy” OR “microalbuminuria” OR “macroalbuminuria” OR “albuminuria” OR “diabetic kidney disease” OR “diabetic neuropathy” OR “neuropathy” OR “sensory neuropathy” OR “peripheral neuropathy” OR “diabetic peripheral neuropathy” OR “diabetic sensorineuropathy” OR “peripheral arterial disease” OR “atherosclerotic vascular disease” OR “diabetic foot” OR “diabetic foot ulcer” OR “foot ulcers” AND “Uganda”.

In addition to the search of the databases above, we also hand-checked the references of the articles whose full texts were retrieved for any additional studies.

### Study selection criteria

The preliminary screening of titles and abstracts to identify potentially eligible articles was done independently by two reviewers (YN and FB) after removing duplicates. In case of any disagreement, an independent reviewer (RO) was consulted.

Full texts of the potentially eligible studies were retrieved and reviewed for the information of interest by three reviewers (DK, WT, and WL). We included randomised controlled trials, cohort, case-control, cross-sectional, and retrospective studies published in English and reporting information of interest, which included: sociodemographic (age, sex, family history of diabetes, and history of current smoking), clinical (duration of diabetes, co-existing HIV, and hypertension, commonly used glucose-lowering therapies, systolic blood pressure [SBP] and diastolic blood pressure [DBP]), anthropometric (body mass index or BMI), and metabolic (fasting blood glucose [FBG] and/or glycated haemoglobin [HbA1c]) characteristics, prevalence, and factors reported to have a statistically significant association with the chronic diabetes complications of interest.

We excluded conference posters, studies not published in English, and studies whose full text could not be retrieved.

### Data extraction

After identifying the eligible studies, the relevant study information of interest was extracted by one author (DK) in May 2024 using a pre-tested data extraction form. All of the extracted data was then transferred into Microsoft Excel. The information that was extracted included: the last name of the first author and year of publication, the number of study participants, the number and proportion of female participants, the proportion of participants with a family history of diabetes, current history of smoking, self-reported co-existing hypertension, and HIV. We also extracted information on the commonly used glucose-lowering treatment (metformin, metformin-sulfonylurea combination, and insulin therapy either as monotherapy or in combination with oral glucose-lowering agents), mean ± standard deviation (SD) or median (interquartile range or IQR) age, duration of diabetes, SBP, DBP, BMI, FBG, HbA1c, prevalence and mode of diagnosis of the diabetes complication of interest, and the associated factors. The five chronic microvascular and macrovascular diabetes complications of interest were diabetic neuropathy, nephropathy, retinopathy, diabetic foot disease (DFD), and peripheral arterial disease (PAD).

### Operational definitions

For the chronic diabetes complications of interest, we considered diagnoses made based on self-report, documentation in medical records, or standard recommended diagnostic approaches. For the latter approach of diagnosing the diabetes complications, we considered the presence of albuminuria (>30 mg/g or 3 mg/mmol) and/or an estimated glomerular filtration rate (e-GFR) of <60 ml/min/1.73 m2 for the diagnosis of diabetic nephropathy [11]. For the diagnosis of diabetic peripheral neuropathy, we considered the presence of suggestive symptoms such as intense pain, paraesthesia, and numbness in the feet, features on neurological examination such as loss of pain and fine touch sensation, reduced ankle reflexes, and the use of neuropathy grading scores such as neuropathy symptom score (NSS) and neuropathy disability score (NDS) [12]. Diabetic retinopathy was diagnosed based on a fundoscopic examination demonstrating the presence of suggestive features such as microaneurysms, retinal dot and blot haemorrhages, hard exudates or cotton wool spots, neovascularisation, vitreous or pre-retinal haemorrhage, retinal detachment, and diabetic macular oedema [13]. The presence of peripheral neuropathy, PAD, infection, ulcer(s), neuro-osteoarthropathy, gangrene, or amputation was suggestive of DFD [14]. A diagnosis of PAD was made based on an ankle-brachial index of <0.90 on arterial Doppler ultrasonography and/or the presence of clinical features such as hyperpigmentation of the skin of the feet, loss of hair, and feeble or absent foot pulses [15].

### Assessment of the quality and publication bias of studies

The quality of all eligible studies included in the systematic review and meta-analysis was assessed using the modified Newcastle-Ottawa Scale (NOS) [16]. This was done independently by one author (APK). Each numbered item in the selection, comparability, and outcome sections of the modified NOS for each study is awarded a score between 0 and 2.

The quality of a study is then determined by summing the total score awarded for all numbered items. The total score ranges from 0 to 10. Very good and good studies were assigned 9–10 points and 7–8 points, respectively. Satisfactory and unsatisfactory studies were studies assigned 5–6 points and 0–4 points, respectively.

### Study outcomes

The study outcomes were the prevalence and factors associated with the five chronic microvascular and macrovascular diabetes complications.

### Data analysis

All analyses were performed using STATA MP 18.0 statistical software (Stata Corp, College Station, Texas, USA). The results were reported in line with PRISMA 2009 guidelines. The descriptive data of all eligible studies were summarised based on the type of variable reported. This included: the participants’ age, sex, family history of diabetes, history of smoking, duration of diabetes, history of comorbidities, such as hypertension and HIV, current glucose-lowering therapies, systolic and diastolic blood pressure, body mass index, fasting blood glucose, and glycated haemoglobin.

For categorical variables, a meta-analysis of proportions was performed using the Freeman-Tukey double arcsine transformation to stabilise variances [17]. We calculated a weighted mean of means for continuous variables reported as means, with weights proportional to study sample sizes. When medians were reported, we pooled the medians by calculating the median of medians. The pooled prevalence of each diabetes complication was determined using a random-effect model meta-analysis of proportions using the Freeman-Tukey double arcsine transformation and presented in the form of forest plots.

The heterogeneity of the included studies was assessed using I^2^ values. Based on the Cochrane collaboration guide, the I^2^ values of 0%–40%, 30%–60%, 50%–90%, and 75%–100% were considered not important, moderate, substantial, and considerable levels of heterogeneity, respectively [18]. We conducted univariate random-effects meta-regression to explore potential sources of heterogeneity and identify study-level covariates that may influence the variability in prevalence estimates across studies.

## Results

The literature search returned a total of 211 articles. From these, 85 duplicates were removed. We reviewed the titles and abstracts of the remaining 126 articles, and 53 articles were identified for full-text retrieval. Of these, 37 were excluded, with 16 articles remaining. The reasons for the exclusion were a lack of information of interest (n = 30 articles), studies with similar baseline information (n = 3 articles), and being a review article (n = 1 article), conference poster (n = 1 article), study protocol (n = 1 article), and pre-print (n = 1 article).

**Fig 1:**
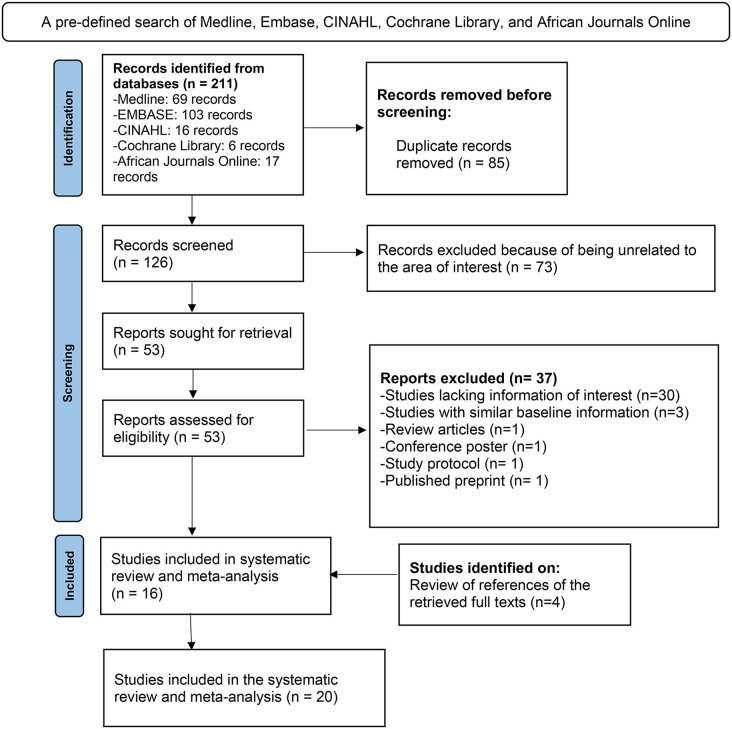
PRISMA flow diagram of selection of eligible studies.

On hand-searching the references of the retrieved full texts, four additional articles were identified to make the total of 20 articles included in this systematic review and meta-analysis) (**Fig 1**)

### Overall characteristics of participants included in this systematic review and meta-analysis

Twenty studies reporting information on a total of 11,400 participants (ranging from 41 to 5,730 participants) were included in this systematic review and meta-analysis. The pooled mean (SD) age, duration of diabetes, and HbA1c of the participants were 54.8 (3.6) years, 6.9 (1.6) years, and 10.5 (2.2) % or 91.0 (1.0) mmol/mol, respectively. About 61.1% (95% confidence interval [CI] 57.1–65.2) of the participants were female. The pooled prevalence of co-existing hypertension and HIV was 51.9% (95% CI 42.8–60.9) and 9.9% (95% CI 5.5–14.2), respectively (**Table 1**).

**Table 1:** Overall characteristics of the participants included in the systematic review and meta-analysis.

Characteristics		Pooled value	Number of studies
**Demographic and clinical**			
Age, years	Mean (SD)	54.8 (3.6)	16
Female participants	%, 95% CI	61.1 (57.1–65.2)	20
Current smoking history	%, 95% CI	9.3 (4.3–14.4)	12
Family history of diabetes	%, 95% CI	41.3 (15.8–66.7)	4
Co-existing HIV	%, 95% CI	9.9 (5.5–14.2)	6
Co-existing hypertension	%, 95% CI	51.9 (42.8–60.9)	13
Insulin therapy (monotherapy or in combination with oral glucose-lowering agents)	%, 95% CI	38.4 (27.2–49.6)	9
Duration of diabetes, years	Mean (SD)	6.9 (1.6)	7
	Median (IQR)	NA	2
Systolic blood pressure, mmHg	Mean (SD)	139.5 (1.5)	3
	Median (IQR)	140 (125–145)	3
Diastolic blood pressure, mmHg	Mean (SD)	84.9 (4.0)	3
	Median (IQR)	81 (80–84)	3
**Anthropometric**			
Body mass index, kg/m^2^	Mean (SD)	26.2 (1.4)	6
	Median (IQR)	NA	2
**Metabolic**			
Fasting blood glucose, mmol/l	Mean (SD)	10.5 (0.8)	3
	Median (IQR)	NA	2
Glycated haemoglobin, %	Mean (SD)	10.5 (2.2)	4
	Median (IQR)	9.1 (8.1–10.6)	3
Glycated haemoglobin, mmol/mol	Mean (SD)	91.0 (1.0)	4
	Median (IQR)	76.0 (65.0-92.0)	3

CI- Confidence interval, IQR- Interquartile range, NA- Not applicable, SD- Standard deviation

#### Characteristics of studies included in this systematic review and meta-analysis.

A total of 20 studies were included in this systematic review and meta-analysis [19–38]. The majority of the studies were cross-sectional in design (17 studies, 85%) [20,21,23–36,38] with only three studies being retrospective [19,22,37]. Of these studies, 15 (75%) were conducted in public hospitals [19–21,24–36] while three (15%) were conducted in private hospitals [22,30,37]. Two studies were conducted in both public and private hospitals [23,38]. Twelve studies (60%) reported the prevalence of chronic diabetes complications based on objective and standardised screening approaches [20,21,23–25,27,28,30,31,33,36,38], while eight studies (40%) based on self-report or the presence of symptoms suggestive of the chronic diabetes complication of interest [19,22,26,29,32,34,35,37] (**Table 2**).

**Table 2:** Characteristics of each study included in the systematic review and meta-analysis.

Last name of 1^st^ author, year of publication, and number of participants	Sociodemographic characteristics	Clinical and anthropometric characteristics	Diabetes complication(s) screened and mode of diagnosis	Prevalence and associated factors of the screened diabetes complication(s)
Alimwenda et al 2022 n = 1000 participants	-Mean age: 51.1 ± 13.1 years -Female participants: 674 (67.4%) -History of current smoking: 20.8%	-Co-existing HIV: 16.9%	-Diabetic neuropathy, nephropathy, foot disease, and retinopathy (all self-reported)	-Diabetic neuropathy: 54.0% -Diabetic nephropathy: 15.6% -Diabetic foot disease: 11.0% -Diabetic retinopathy: 9.0%
Arunga et al 2020 n = 5,730 participants	-Median (IQR) age: 56.0 (46.0–100.0) years -Female participants: 4,189 (73.1%)		-Diabetic retinopathy (diagnosed based on findings of retinal photography using a portable non-mydriatic fundus camera)	-Diabetic retinopathy: 5.1%
Bateganya et al 2003 n = 120 participants	-Mean age: 49.0 ± 18.0 years -Female participants: 69 (57.5%) -History of current smoking: 11.7%	-Co-existing HT: 36.7% -Participants on insulin therapy: 47.5%	-Diabetic neuropathy (diagnosed based on the presence of suggestive symptoms and clinical examination) -Diabetic nephropathy (diagnosed based on the presence of proteinuria) -Diabetic foot disease (diagnosed based on clinical examination)	-Diabetic neuropathy: 38.5% -Diabetic nephropathy: 25.8% -Diabetic foot disease: 3.3%
Kibirige et al 2014 n = 250 participants	-Mean age: 51.6 ± 9.2 years -Female participants: 155 (62.0%) -Family history of diabetes: 37.2%-History of current smoking: 1.2%	-Mean BMI: 24.5 ± 3.9 kg/m^2^ -Co-existing HT: 78.4% -Participants on metformin and sulfonylureas: 71.2% -Participants on metformin only: 28.8% -Participants on insulin therapy: 20%	-Diabetic neuropathy, nephropathy, retinopathy, foot disease, PAD, and ischaemic heart disease (all self-reported)	-Diabetic neuropathy: 31.2% -Diabetic retinopathy: 9.6% -Diabetic foot disease: 3.2% -PAD: 2.0% -Ischaemic heart disease: 1.2% -Diabetic nephropathy: 0.8%
Kibirige et al 2023 n = 519 participants	-Median (IQR) age: 48 (39–57) years -Female participants: 292 (56.3%)	-Co-existing HT: 33.7% -Median (IQR) SBP: 125 (115–136) mmHg -Median (IQR) DBP: 84 (77–91) mmHg -Median (IQR) BMI: 27.4 (23.4–31.4) mmHg -Median (IQR) FBG: 8.6 (6.3–13.4) mmol/l -Median (IQR) HbA1c: 10.6 (7.8–12.5) % or 92.0 (62.0–113.0) mmol/mol	-Diabetic nephropathy (diagnosed based on the presence of albuminuria)	-Diabetic nephropathy: 33.7%. Associated factors -Self-reported HT comorbidity (AOR: 1.76, 95% CI 1.24–2.48, p = 0.002) -BMI ≥ 30 kg/m^2^ (AOR: 0.61, 95% CI 0.41–0.91, p = 0.02)
Kiconco et al 2019 n = 140 participants	-Female participants: 95 (67.9%) -Family history of diabetes: 77.9% -History of current smoking: 2.1%	-Mean duration of diabetes: 6.8 years -Co-existing HT: 42.1% -Mean SBP: 138.6 mmHg -Mean DBP: 78.1 mmHg -Mean BMI: 24.4 kg/m^2^ -Mean ± SD FBG: 9.3 ± 5.4 mmol/l	-Diabetic nephropathy (diagnosed based on the presence of microalbuminuria)	-Diabetic nephropathy: 23.6% Associated factors Family history of diabetes (β = 0.275, 95% CI 0.043–0.508, p = 0.002)
Kisozi et al 2017 n = 248 participants	-Mean age: 48.5 ± 13.4 years -Female participants: 94 (37.9%)		-Diabetic neuropathy (diagnosed based on the presence of suggestive symptoms and the NSS and NDS)	-Diabetic neuropathy: 29.4% Associated factors -Age > 60 years (AOR: 3.72, 95%CI 1.25–11.03, p = 0.018) -History of a foot ulcer (AOR- 2.59, 95% CI 1.03–6.49, p = 0.042)
Lumori et al 2022 n = 195 participants	-Mean age: 62.0 ± 11.5 years -Female participants: 141 (72.3%) -Current history of smoking: 0.5%	-Median (IQR) duration of diabetes: 10.0 (7.0–15.0) years -Co-existing HT: 74.9% -Co-existing HIV: 7.2% -Median (IQR) SBP: 145 (128–158) mmHg - Median (IQR) DBP: 81 (75–89) mmHg -Median (IQR) HbA1c: 9.1 (7.7–10.9) % or 76.0 (61.0–96.0) mmol/mol	-Diabetic neuropathy (diagnosed based on the presence of suggestive symptoms)	-Diabetic neuropathy: 61.0%
Magan et al 2019 n = 41 participants	-Mean ± SD age: 50.4 ± 12.5 years -Female participants: 26 (63.4%)	-Mean ± SD duration of diabetes: 8.1 ± 6.4 years -Co-existing HT: 65.9% -Participants on insulin therapy: 51.2%	-Diabetic retinopathy (diagnosed based on findings of indirect fundoscopy ± retinal photography using a portable non-mydriatic fundus camera)	-Diabetic retinopathy: 19.5%
Migisha et al 2020 n = 299 participants	-Mean ± SD age: 50.1 ± 9.8 years -Female participants: 208 (69.6%) -Current history of smoking: 25.7%	-Mean ± SD duration of diabetes: 5.8 ± 5.9 years -Mean ± SD BMI: 27.4 ± 5.6 kg/m2 -Mean ± SD SBP: 141.7 ± 22.6 mmHg -Mean ± SD DBP: 87.1 ± 10.7 mmHg -Mean ± SD FBG: 11.2 ± 4.8 mmHg -Mean ± SD HbA1c: 9.7 ± 2.6% or 83.0 ± 5.0 mmol/mol	-Diabetic neuropathy and retinopathy (all self-reported)	-Diabetic neuropathy: 61.2% -Diabetic retinopathy: 22.7%
Muddu et al 2018 n = 175 participants	-Mean ± SD age: 46.0 ± 15.0 years -Female participants: 85 (48.6%)	-Mean ± SD HbA1c: 13.9 ± 5.3% or 128.0 ± 34.0 mmol/mol	-Diabetic nephropathy (diagnosed based on the presence of microalbuminuria)	-Diabetic nephropathy: 47.4% Associated factors -Pregnancy (AOR: 7.74, 95% CI 1.01–76.47, p = 0.05) -Mild and moderate physical activity (AOR: 0.08, 95% CI 0.01–0.95, p = 0.046)
Munyambalu et al 2023 n = 319 participants	-Mean age: 59.4 ± 14.6 years -Female participants: 197 (61.8%) -History of current smoking: 13.5%	-Mean ± SD duration of diabetes: 7.3 ± 6.4 years -Co-existing HT: 50.2% -Co-existing HIV: 9.1% -Participants on insulin therapy: 12.9% -Mean ± SD BMI: 26.3 ± 3.5 years -Mean ± SD SBP: 138.4 ± 19.8 mmHg -Mean ± SD DBP: 85.8 ± 13.0 mmHg -Mean ± SD FBG: 10.4 ± 5.2 mmol/l	-Diabetic neuropathy (diagnosed based on the presence of suggestive symptoms and the NDS)	-Diabetic neuropathy: 65.8%
Mwebaze et al 2014 n = 146 participants	-Mean ± SD age: 53.9 ± 12.4 years -Female participants: 71 (48.6%) -History of current smoking: 0.7%	-Mean ± SD duration of diabetes: 5.8 ± 3.2 years -Co-existing HT: 47.3% -Participants on insulin therapy: 36.7% -Mean ± SD HbA1c: 9.5 ± 2.5% or 80.0 ± 4.0 mmol/mol	-PAD (diagnosed based on an ABI ≤ 0.90 on Doppler ultrasonography)	-PAD: 39%
Nambuya et al 1996 n = 252 participants	-Mean age: 45 years -Female participants: 135 (53.6%) -Family history of diabetes: 16.3%	-Co-existing HT: 27.3% -Participants on insulin therapy: 52.8%	-Diabetic neuropathy, nephropathy, foot disease, and ischaemic heart disease (all self-reported)	-Diabetic neuropathy: 46.4% -Diabetic nephropathy: 4.8% -Ischaemic heart disease: 4.8% -Diabetic foot disease: 4.0%
Okello et al 2014 n = 229 participants	-Median (IQR) age: 60 (55–66) years -Female participants: 146 (63.7%) -Family history of diabetes: 34.1% -History of current smoking: 5.2%	-Median (IQR) duration of diabetes: 1.0 (0.3–2.3) years -Co-existing HT: 49.3% -Co-existing HIV: 7.9% -Participants on metformin only: 51.6% -Participants on metformin and glibenclamide: 15.7% -Participants on insulin therapy: 29.3% -Median (IQR) SBP: 140 (124–160) mmHg -Median (IQR) DBP: 80 (70–90) mmHg -Median (IQR) FBG: 8.6 (6.6–13.7) mmol/l -Median (IQR) HbA1c: 8.1 (6.7–10.1) % or 65.0 (50.0–87.0) mmol/mol	-PAD (diagnosed based on an ABI ≤ 0.90 on doppler ultrasonography)	-PAD: 24% Associated factors -Female sex (AOR: 2.25, 95% CI 1.06–4.77, p = 0.034) -Current high blood pressure (AOR: 2.59, 95% CI 1.25–5.33, p = 0.01) -Using glibenclamide (AOR: 3.47, 95% CI 1.55–7.76, p = 0.002)
Pario et al 2022 n = 60 participants	-Female participants: 42 (70%)	-Participants on insulin therapy: 33.3%	-Diabetic neuropathy (diagnosed based on the presence of suggestive symptoms) -PAD (diagnosed based on the presence of symptoms of claudication and rest pain)	-Diabetic neuropathy: 91.7% -PAD: 65%
Patrick et al 2021 n = 223 participants	-Mean age: 61.1 ± 4.0 years -Female participants: 153 (68.9%) -History of current smoking: 2.2%	-Mean duration of diabetes: 6.1 ± 7.1 years -BMI: 27.9 ± 5.2 kg/m^2^	-Diabetic neuropathy and retinopathy (all self-reported)	-Diabetic neuropathy: 85.7% -Diabetic retinopathy: 65.1%
Sikhondze et al 2022 n = 62 participants	-Mean ± SD age: 57.0 ± 12.3 years -Female participants: 35 (56.5%) -History of current smoking: 22.6%	-Co-existing HT: 40.3% -Co-existing HIV: 17.7%	-PAD (diagnosed based on an ABI ≤ 0.90 on Doppler ultrasonography) -Diabetic nephropathy and retinopathy (self-reported)	-PAD: 35.5% -Diabetic retinopathy: 6.5% -Diabetic nephropathy: 3.2%
Tino et al 2020 n = 1,275 participants	-Median (IQR) age: 54 (44–65) years -Female participants: 770 (60.4%) -History of current smoking: 10.9%	-Co-existing HT: 69.6%	-Diabetic neuropathy, nephropathy, and PAD (all self-reported)	-Diabetic neuropathy: 37.7% -Diabetic nephropathy: 6.4% -PAD: 2.3%
Vahwere et al 2023 n = 117 participants	-Mean age: 57.1 years -Female participants: 72 (61.5%)	-Mean ± SD duration of diabetes: 10.9 ± 9.7 years -Co-existing HT: 59% -Participants on insulin therapy: 65.0% -Mean ± SD BMI: 25.3 ± 5.9 mmol/l -Mean ± SD HbA1c: 8.6 ± 2.6% or 70.0 ± 5.0 mmol/mol	-Diabetic neuropathy (diagnosed based on the presence of suggestive symptoms) -PAD (diagnosed based on the presence of symptoms of claudication, features of gangrene, and an ABI ≤ 0.90 on Doppler ultrasonography)	-Diabetic neuropathy: 79.5% -PAD: 30.7%

ABI- Ankle brachial index, AOR- Adjusted odds ratio, BMI- Body mass index, CI- Confidence interval, DBP- Diastolic blood pressure, FBG- Fasting blood glucose, HbA1c- Glycated haemoglobin, HT- Hypertension, IQR- Interquartile range, NDS- Neuropathy disability score, NSS- Neuropathy symptom score, PAD- Peripheral arterial disease, SBP- Systolic blood pressure, SD- Standard deviation

### Assessment of study quality

Based on the modified NOS, all studies were of low quality. Of the 20 studies, 14 (70%) were considered satisfactory, while the remaining six studies were considered unsatisfactory (**S2 Table**).

#### Prevalence of chronic microvascular and macrovascular diabetes complications.

Regarding chronic microvascular diabetes complications, information on the prevalence of diabetic neuropathy, nephropathy, and retinopathy was reported by 12 studies [19,21,22,25,26,29,30,32,34,35,37,38], nine studies [19,21–24,28,32,36,37], and seven studies [19,20,22,27,35–37], respectively. For chronic macrovascular diabetes complications, information on the prevalence of PAD and DFD was reported by six studies [22,31,33,34,36,38] and four studies [19,21,22,32], respectively.

#### Pooled prevalence of diabetic neuropathy.

**Fig 2:**
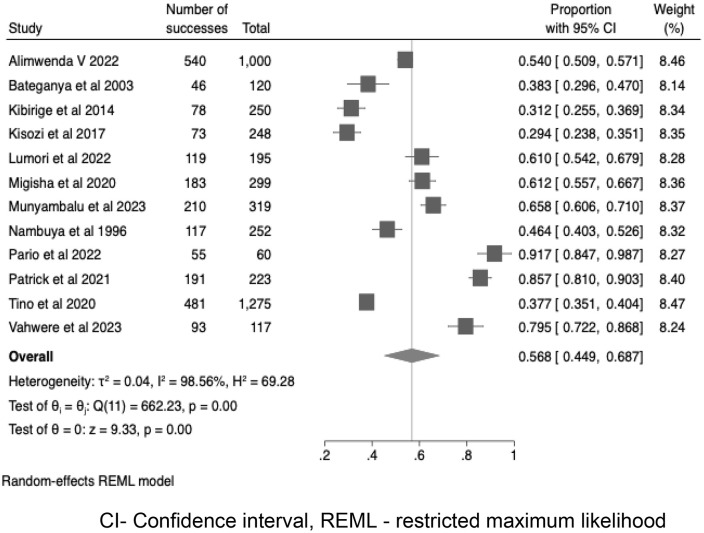
The pooled prevalence of diabetic neuropathy.

Of the 12 studies, the prevalence of diabetic neuropathy was based on self-report (presence of symptoms suggestive of peripheral neuropathy only) in seven studies (58.3%) [19,22,26,32,35,37,38]. The remaining five studies diagnosed diabetic neuropathy based on the presence of suggestive symptoms, a neurological clinical examination, and the use of neuropathy grading scores such as NDS and NSS [21,25,29,30,34]. The pooled prevalence of diabetic neuropathy in the 12 studies was 56.8% (95% CI 44.9–68.7, I^2^ = 98.56%, p < 0.001) (**Fig 2**)

#### Pooled prevalence of diabetic retinopathy.

**Fig 3:**
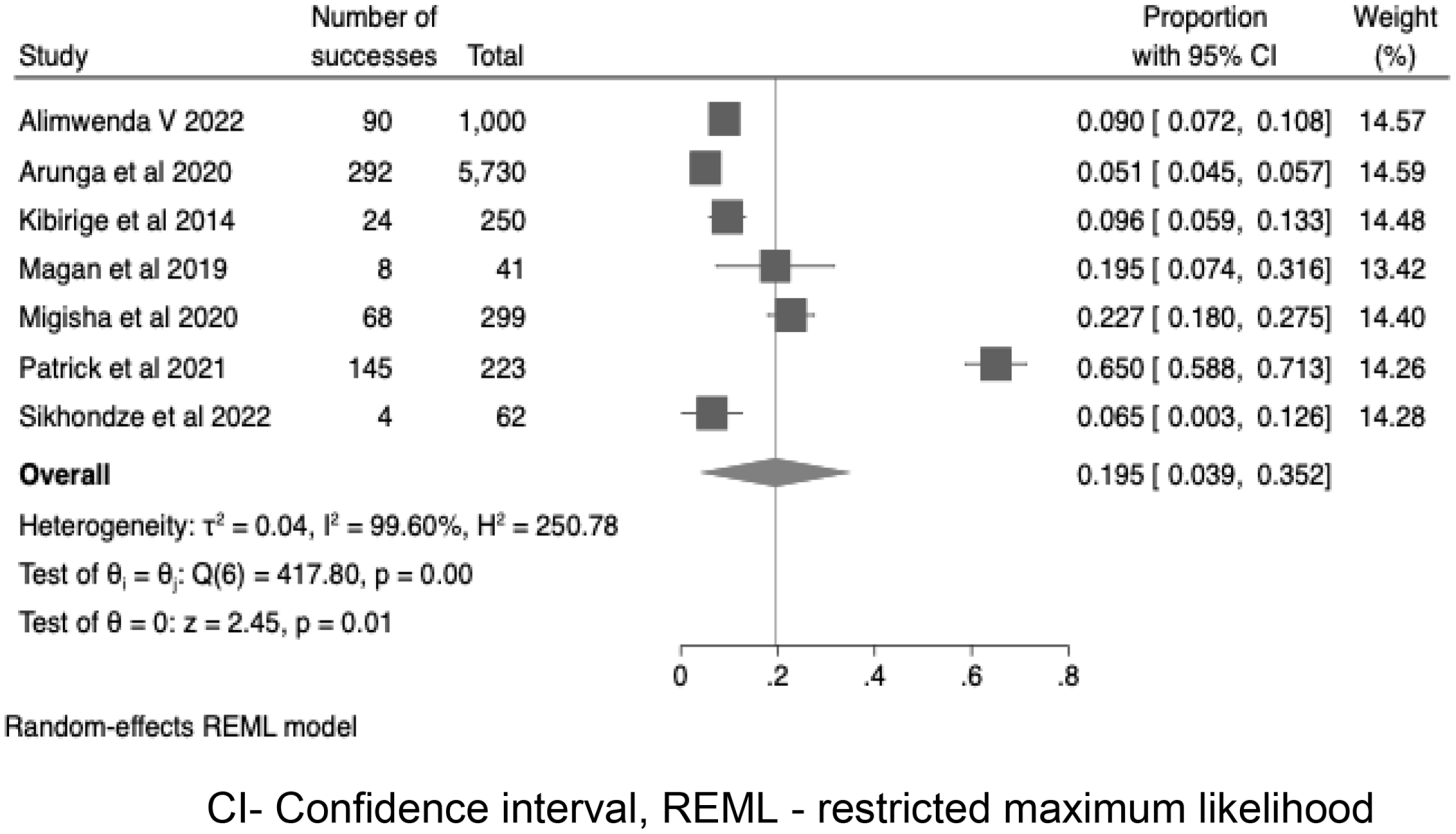
The pooled prevalence of diabetic retinopathy.

Of the seven studies reporting information on the prevalence of diabetic retinopathy, only two studies (28.6%) made the diagnosis based on fundoscopic examination [20,27]. The rest of the studies based the diagnosis on self-report [19,22,35–37]. The pooled prevalence of diabetic retinopathy in the seven studies was 19.5% (95% CI 3.9–35.2, I^2^ = 99.60%, p < 0.001) (**Fig 3**)

#### Pooled prevalence of diabetic nephropathy.

**Fig 4:**
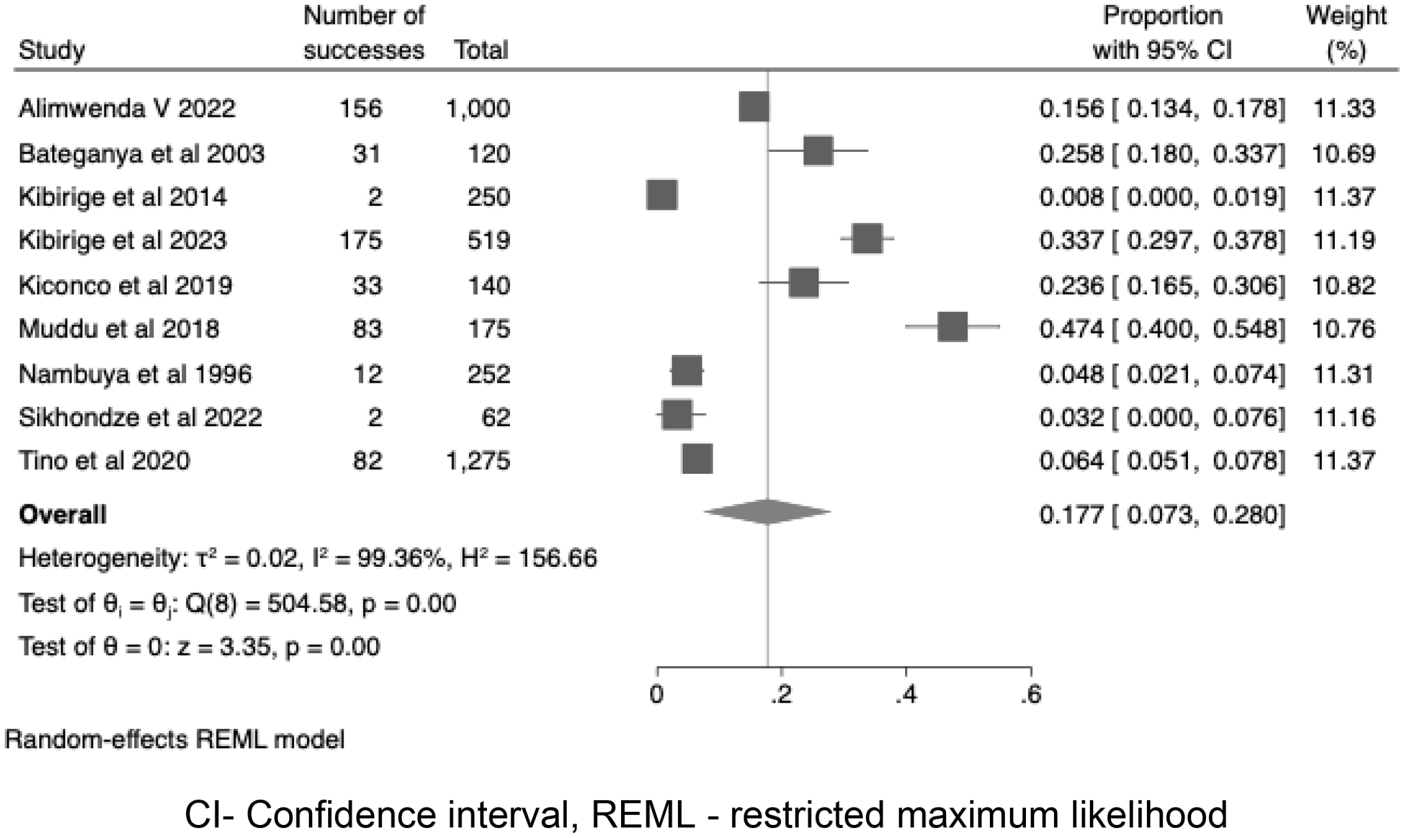
The pooled prevalence of diabetic nephropathy.

Among the nine studies that reported the prevalence of diabetic nephropathy, five (55.6%) based the diagnosis on the presence of albuminuria [21,23,24,28,32]. The diagnosis in the remaining four studies was based on self-report [19,22,36,37]. The pooled prevalence of diabetic nephropathy in these studies was 17.7% (95% CI 7.3–28.0, I^2^ = 99.36%, p < 0.001) (**Fig 4**)

#### Pooled prevalence of peripheral arterial disease and diabetic foot disease.

**Fig 5:**
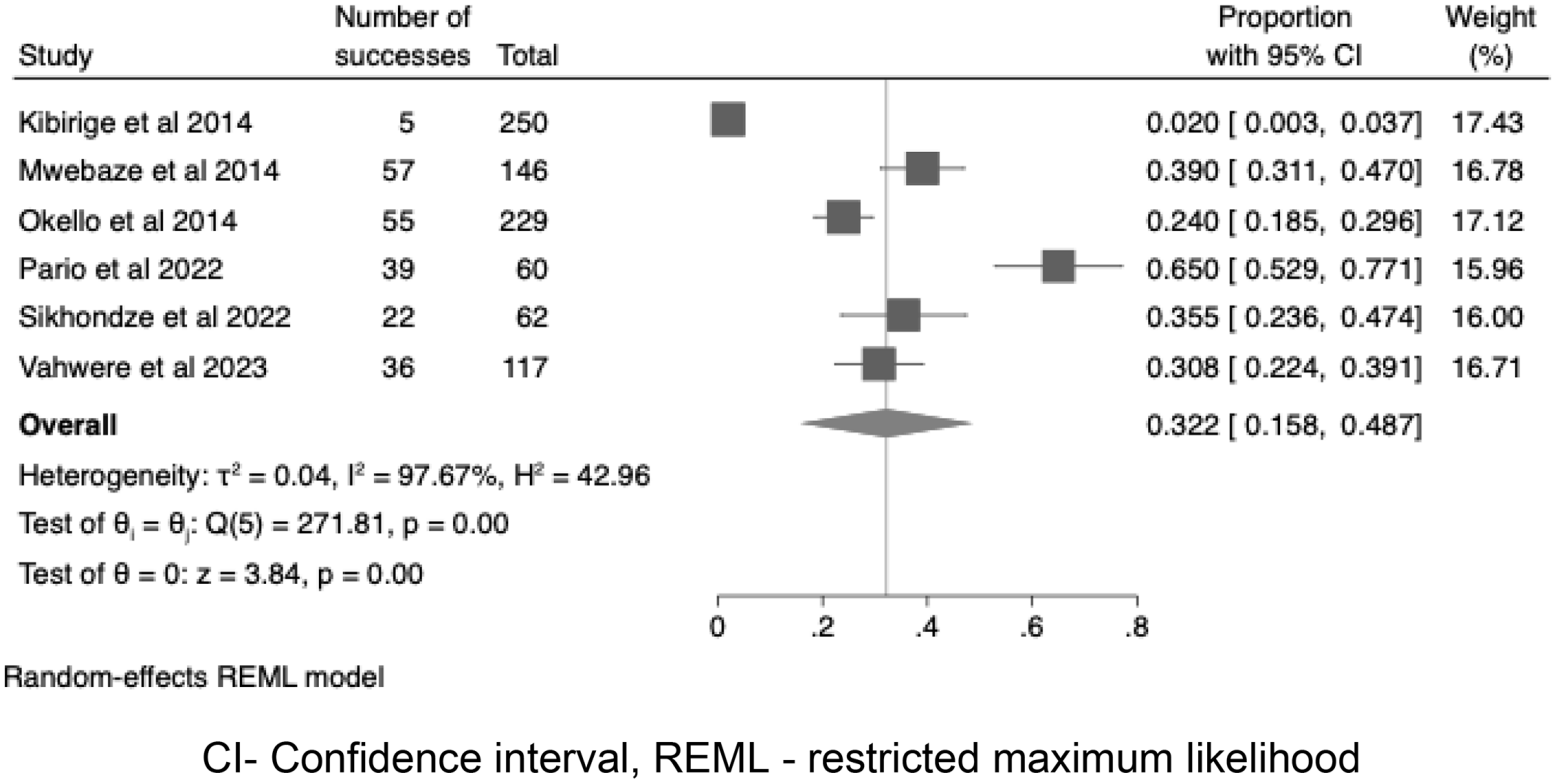
The pooled prevalence of peripheral arterial disease.

For the macrovascular diabetes complications, regarding the prevalence of PAD reported by the six studies, the diagnosis was made based on clinical examination and/or Doppler ultrasonography in five studies (83.3%) [31,33,34,36,38], with one study basing the diagnosis on self-report [22]. The pooled prevalence of PAD was 32.2% (95% CI 15.8–48.7, I^2^ = 97.67%, p < 0.001) (**Fig 5**)

**Fig 6:**
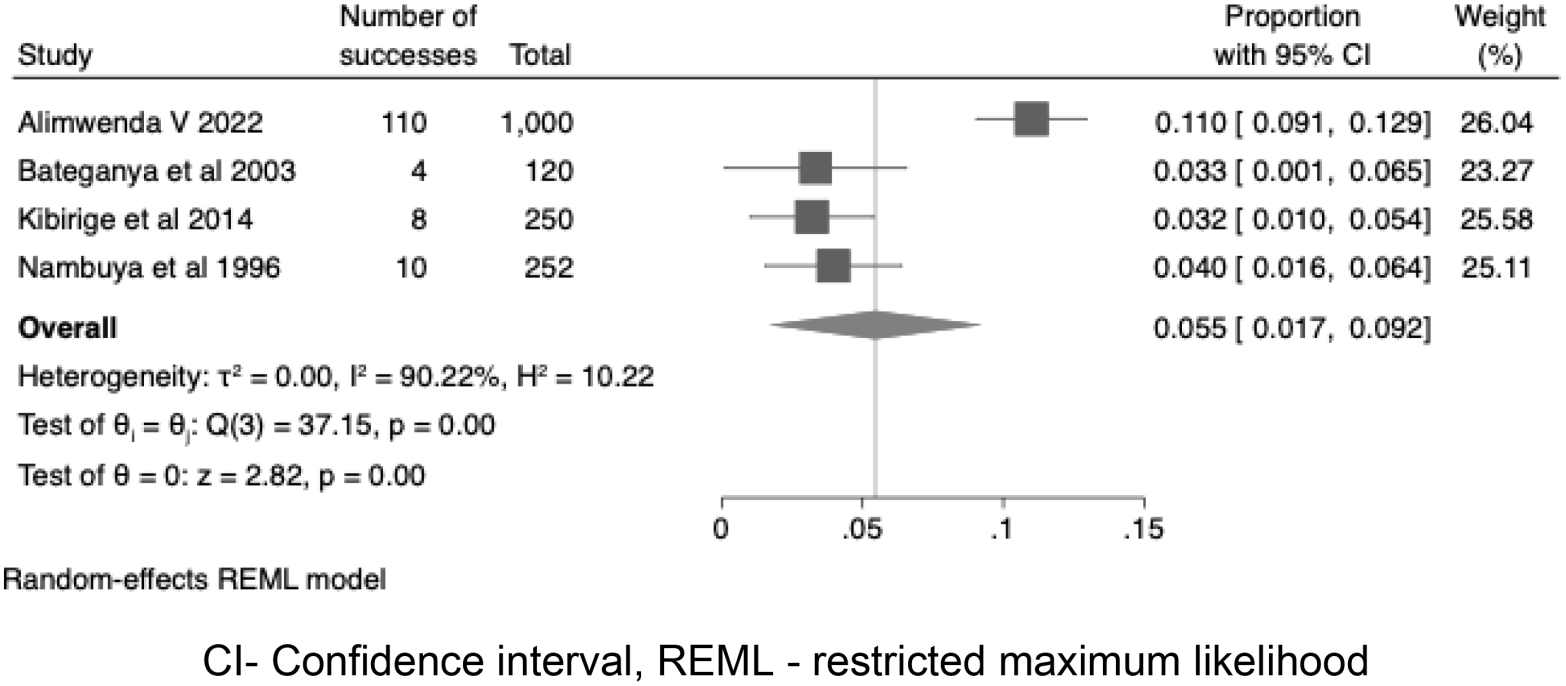
The pooled prevalence of diabetic foot disease.

The diagnosis of DFD was based on clinical examination in all four studies [19,21,22,32], documenting a pooled prevalence of 5.5% (95% CI 1.7–9.2, I^2^ = 90.22%, p < 0.001) (**Fig 6**)

### Factors significantly associated with the five chronic microvascular and macrovascular diabetes complications

The information on the factors significantly associated with the five chronic diabetes complications was reported by only five studies (25%) [23–25,28,33]. Of these, three studies reported associated factors of diabetic nephropathy [23,24,28] while the remaining two studies reported associated factors of diabetic neuropathy [25] and PAD [33].

### Factors significantly associated with diabetic nephropathy

The factors significantly associated with diabetic nephropathy in the three studies were self-reported hypertension comorbidity (adjusted odds ratio [AOR]: 1.76, 95% CI 1.24–2.48, p = 0.002), pregnancy (AOR: 7.74, 95% CI 1.01–76.47, p = 0.05), and family history of diabetes (β = 0.275, 95% CI 0.043–0.508, p = 0.002) which were associated with increased odds of having diabetic nephropathy, while BMI ≥ 30 kg/m^2^ (AOR: 0.61, 95% CI 0.41–0.91, p = 0.02) and mild to moderate physical activity (AOR: 0.08, 95% CI 0.01–0.95, p = 0.046) were associated with reduced odds [23,24,28].

### Factors significantly associated with diabetic neuropathy and peripheral arterial disease

In the study by Kisozi et al, the factors significantly associated with diabetic neuropathy were a history of a foot ulcer (AOR: 2.59, 95% CI 1.03–6.49, p = 0.042) and age > 60 years (AOR: 3.72, 95% CI 1.25–11.03, p = 0.018) [25]. The factors significantly associated with PAD in the study by Okello et al were female sex (AOR: 2.25, 95% CI 1.06–4.77, p = 0.034), current hypertension (AOR: 2.59, 95% CI 1.25–5.33, p = 0.01), and being on glibenclamide (AOR: 3.47, 95% CI 1.55–7.76, p = 0.002) [33].

### Meta-regression analysis

The meta-regression models explored the influence of various covariates, such as age, proportion of females, smoking status, co-existing HIV, insulin therapy, hypertension, and BMI, on the proportion of individuals affected by these complications (**S3 Table****)**.

For diabetic neuropathy, the mean age and proportion of females were found to be significant predictors of neuropathy prevalence. Each unit increase in mean age in years was associated with a 2.34% increase in the prevalence of neuropathy (coefficient: 0.0234; 95% CI: 0.0079, 0.0388; p = 0.003). The model explained 47.6% heterogeneity (R² = 47.6%). Similarly, each percentage increase in the proportion of females in the study was associated with a 1.41% increase in the prevalence of neuropathy (coefficient: 0.0141; 95% CI: 0.0034, 0.0249; p = 0.010, R² = 34.8%). No other variables, including smoking status, coexisting HIV, insulin usage, and hypertension, were significant associated factors (p > 0.05).

For PAD, the proportion of patients with hypertension was the only significant predictor, with each unit increase in hypertension prevalence associated with a 0.92% decrease in PAD prevalence (coefficient: −0.0092; 95% CI: −0.0142, −0.0041; p = 0.000), explaining 77.64% of the variance. Other variables, including mean age, proportion of females, smoking status, and insulin usage, were not statistically significant (p > 0.05).

For all other diabetes complications, no significant factors were found to be associated with their prevalence.

## Discussion

In this systematic review and meta-analysis, we report a high prevalence of chronic diabetes complications in adult Ugandans with diabetes. Diabetic neuropathy and PAD are the most prevalent in this study population.

Comparing our study results with the findings of several studies, systematic reviews, and meta-analyses on the prevalence of diabetes complications in other adult African populations, diabetic neuropathy is also reported as the most prevalent chronic diabetes complication [3,39–44]. In one systematic review and meta-analysis of 23 studies describing 269,691 Africans with diabetes by Shiferaw et al, a comparably high prevalence of diabetic neuropathy of 46% was reported [44].

Regarding the prevalence of diabetic retinopathy, several studies conducted in Nigeria [45], Botswana [46], Ethiopia [47–49], and South Africa [50] have reported a comparable prevalence ranging between 17.7% to 21%.

The prevalence of diabetic nephropathy in adult Africans reported by some systematic reviews and meta-analyses varies widely in comparison to our study findings. One systematic review and meta-analysis of studies assessing the burden of chronic microvascular complications in Ethiopia reported a prevalence of diabetic nephropathy comparable to what we observed in our study (11.5%) [49]. In another systematic review and meta-analysis of 19 studies investigating the burden of diabetic nephropathy in Nigeria, a higher prevalence of 28% was observed [51], compared with our study (17.7%).

Generally, a high prevalence of diabetic nephropathy, ranging from 21.0% to 35.3% has been reported in adult African populations with diabetes in several systematic reviews and meta-analyses [3,42,52,53]. This heterogeneity in the prevalence may be explained by differences in study definitions of diabetic nephropathy (self-reported vs. diagnosed based on assessment for albuminuria and/or e-GFR).

Regarding macrovascular diabetes complications, a high prevalence of PAD, similar to what we observed in our study, has been reported in adult Africans with diabetes [54–57]. Studies conducted in Nigeria [55], Ethiopia [56], and Egypt [57] reported a high comparable prevalence of PAD ranging between 30.7% to 38.5%.

As observed in our study, DFD has been reported as the least prevalent diabetes complication in adult Africans with diabetes in systematic reviews and meta-analyses. Kibirige et al [3] and Rigato et al [43] reported a prevalence of DFD in large adult African populations of 11% and 13%, respectively.

There are several plausible explanations for the high prevalence of these chronic diabetes complications in adult Ugandans with diabetes. The rate of undiagnosed diabetes remains very high, with the majority of people presenting late with diabetes complications [5,6]. Glycaemic, blood pressure, lipid screening, management, and monitoring in most adult Ugandans with diabetes remain largely suboptimal [22,58,59]. This significantly increases the risk of onset and progression of chronic diabetes complications.

Additionally, Uganda still grapples with the challenges of low access to affordable essential diabetes medicines and diagnostic tests, gaps in knowledge in preventing and managing diabetes among healthcare professionals and individuals living with diabetes, and a poorly structured healthcare system to manage chronic conditions such as diabetes [8,9,59–62].

Regarding the factors significantly associated with the five diabetes complications, consistent with findings from other studies, co-existing hypertension [42,52] and reduced physical activity [63] have been associated with increased odds of developing diabetic nephropathy.

While obesity has been widely reported to increase the risk of diabetic nephropathy in most populations [64], it was associated with a reduced risk in one of the studies that we included in the meta-analysis [23]. A similar protective effect of obesity towards developing diabetic nephropathy was also reported in a study conducted in rural South Africa. In this study, severe obesity (defined as BMI > 33 kg/m^2^) reduced the risk of developing microalbuminuria by 73% [65]. This demonstrates differences in some of the risk factors for diabetic nephropathy across populations.

Similar to the Ugandan study by Kisozi et al [25], one study conducted on adult Ethiopians with type 2 diabetes reported an association between age > 60 years and the presence of diabetic neuropathy [66].

Additionally, as reported in another Ugandan study by Okello et al [33], hypertension was identified as one of the associated factors of PAD in a Southern African [67] and Central African population [68].

### Strengths and limitations

To our knowledge, this is the first systematic review and meta-analysis to document the prevalence of microvascular and macrovascular diabetes complications in adult Ugandans with diabetes. The studies were conducted in different regions of Uganda, improving the generalisability of the results of the systematic review and meta-analysis.

Despite these strengths, the systematic review and meta-analysis had some limitations. Some of the included studies used self-reporting as the basis for diagnosing the chronic diabetes complication of interest. This is subject to recall bias and reporting imprecise results. There was considerable heterogeneity in the study sample sizes, study findings, possibly due to the differences in screening procedures, study definitions for the complications of interest, and study settings (public vs. private healthcare facilities). The studies included in this systematic review and meta-analysis were of low-quality rating, with some studies missing important sociodemographic and clinical characteristics.

### Conclusions

This systematic review and meta-analysis reported a high prevalence of microvascular and macrovascular diabetes complications in adult Ugandans with diabetes. Diabetic neuropathy and PAD are the most prevalent chronic diabetes complications.

The clinical implications of these study findings are several. Because of the high prevalence, healthcare practitioners in Uganda should be encouraged to routinely screen for these chronic diabetes complications in every individual with diabetes at the time of diagnosis and during routine clinical care. Optimal and timely management of these chronic diabetes complications should also be encouraged to prevent progression and related mortality.

In clinical settings with resource constraints, a targeted screening approach for these chronic diabetes complications should be adopted. Healthcare practitioners in Uganda should be encouraged to screen for: 1) diabetic nephropathy in people with co-existing hypertension, a family history of diabetes, a sedentary lifestyle, and a non-obese phenotype; 2) diabetic neuropathy in people over the age of 60 and those with foot ulcers; and 3) peripheral arterial disease in women, people with co-existing hypertension, and people taking glibenclamide as a glucose-lowering therapy.

Despite the low rates of smoking and obesity in this study population, the prevalence of PAD, as reported by this systematic review and meta-analysis, is high. The plausible mechanisms to explain this clinical observation need to be investigated further in this study population.

## Supporting information

S1 Table PRISMA checklist. (DOCX)

S2 Table Assessment of the quality of studies using the modified Newcastle Ottawa scale. (DOCX)

S3 Table Meta-regression model evaluating the association between specific characteristics and diabetes complications. (DOCX)
