# Supplementary material for: Prevalence and factors associated with microvascular and macrovascular diabetes complications in adult Ugandans: A systematic review and meta-analysis
Source: PLoS One. 2026 Jun 8;21(6):e0312792. doi: 10.1371/journal.pone.0312792 (PMC13245771; doi:10.1371/journal.pone.0312792)
Supplement: S3 Table — (DOCX) [file pone.0312792.s003.docx]

**S3 Table. The meta-regression model evaluating the association between specific characteristics and diabetes complications**

| **Variable** | **Coefficient (95% CI)** | **p-value** | **R² (%)** | **I² (%)** | **N** |
| --- | --- | --- | --- | --- | --- |
| **Neuropathy** |  |  |  |  |  |
| Mean age | 0.0234 (0.0079, 0.0388) | **0.003** | 47.6% | 96.1% | 10 |
| Mean duration of diabetes | 0.0155 (-0.0498, 0.0807) | 0.642 | 0.0% | 96.1% | 4 |
| Proportion of females | 0.0141 (0.0034, 0.0249) | **0.010** | 34.8% | 97.8% | 12 |
| Current smokers | -0.0001 (-0.0156, 0.0155) | 0.995 | 0.0% | 98.6% | 8 |
| Coexisting HIV | 0.0073 (-0.0193, 0.0340) | 0.590 | 0.0% | 94.4% | 4 |
| Insulin alone or in combination | 0.0017 (-0.0099, 0.0133) | 0.771 | 0.0% | 98.4% | 6 |
| Proportion of hypertension | -0.0003 (-0.0083, 0.0076) | 0.934 | 0.0% | 97.9% | 7 |
| Mean BMI | 0.0944 (-0.0365, 0.2252) | 0.157 | 20.7% | 97.7% | 5 |
| Mean HbA1c | — | — | — | — | 2 |
| **Nephropathy** |  |  |  |  |  |
| Mean age | -0.0200 (-0.0548, 0.0148) | 0.260 | 4.4% | 99.3% | 6 |
| Mean duration of diabetes | — | — | — | — | 0 |
| Proportion of females | -0.0091 (-0.0267, 0.0085) | 0.312 | 0.0% | 99.4% | 9 |
| Current smokers | -0.0010 (-0.0124, 0.0104) | 0.863 | 0.0% | 98.7% | 6 |
| Coexisting HIV | -0.0120 (-0.0267, 0.0027) | 0.110 | 46.2% | 95.3% | 3 |
| Insulin alone or in combination | 0.0037 (-0.0091, 0.0165) | 0.569 | 0.0% | 96.3% | 3 |
| Proportion of hypertension | -0.0034 (-0.0085, 0.0018) | 0.204 | 9.2% | 99.0% | 7 |
| Mean BMI | — | — | — | — | 3 |
| Mean HbA1c | — | — | — | — | 1 |
| **Retinopathy** |  |  |  |  |  |
| Mean age | 0.0282 (-0.0085, 0.0649) | 0.132 | 17.8% | 99.1% | 7 |
| Mean duration of diabetes | -0.0908 (-0.4495, 0.2679) | 0.620 | 0.0% | 99.2% | 3 |
| Proportion of females | 0.0100 (-0.0219, 0.0420) | 0.538 | 0.0% | 99.4% | 7 |
| Current smokers | -0.0100 (-0.0305, 0.0105) | 0.339 | 0.0% | 99.3% | 5 |
| Coexisting HIV | — | — | — | — | 2 |
| Insulin alone or in combination | — | — | — | — | 2 |
| Proportion of hypertension | 0.0012 (-0.0042, 0.0067) | 0.660 | 0.0% | 65.8% | 3 |
| Mean BMI | 0.1223 (-0.0722, 0.3167) | 0.218 | 20.6% | 98.9% | 3 |
| Mean HbA1c | — | — | — | — | 1 |
| **Diabetic foot ulcers** |  |  |  |  |  |
| Mean age | 0.0044 (-0.0122, 0.0210) | 0.602 | 0.0% | 92.3% | 4 |
| Mean duration of diabetes | — | — | — | — | 0 |
| Proportion of females | 0.0049 (-0.0009, 0.0107) | 0.098 | 40.4% | 83.5% | 4 |
| Current smokers | 0.0039 (-0.0005, 0.0083) | 0.084 | 54.0% | 80.2% | 3 |
| Coexisting HIV | — | — | — | — | 2 |
| Insulin alone or in combination | 0.0002 (-0.0007, 0.0011) | 0.691 | 0.0% | 0.2% | 3 |
| Proportion of hypertension | -0.0001 (-0.0007, 0.0005) | 0.687 | 0.0% | 0.1% | 3 |
| Mean BMI | — | — | — | — | 1 |
| Mean HbA1c | — | — | — | — | 0 |
| **Peripheral artery disease** |  |  |  |  |  |
| Mean age | 0.0208 (-0.0254, 0.0670) | 0.377 | 0 | 93.7% | 5 |
| Mean duration of diabetes | — | — | — | — | 2 |
| Proportion of females | 0.0051 (-0.0226, 0.0327) | 0.719 | 0 | 98.2% | 6 |
| Current smokers | 0.0067 (-0.0144, 0.0279) | 0.532 | 0 | 98.0% | 4 |
| Coexisting HIV | — | — | — | — | 2 |
| Insulin alone or in combination | 0.0037 (-0.0108, 0.0183) | 0.615 | 0 | 98.4% | 5 |
| Proportion of hypertension | -0.0092 (-0.0142, -0.0041) | **0.000** | 77.64% | 75.6% | 5 |
| Mean BMI | — | — | — | — | 2 |
| Mean HbA1c | — | — | — | — | 2 |

BMI- Body mass index, HbA1c- Glycated haemoglobin
